# HIF Stabilization Weakens Primary Cilia

**DOI:** 10.1371/journal.pone.0165907

**Published:** 2016-11-03

**Authors:** Andrew Resnick

**Affiliations:** 1 Department of Physics, Cleveland State University, Cleveland, Ohio, United States of America; 2 Center for Gene Regulation in Health and Disease, Cleveland State University, Cleveland, Ohio, United States of America; Center for Molecular Biotechnology, ITALY

## Abstract

Although solitary or sensory cilia are present in most cells of the body and their existence has been known since the sixties, very little is known about their functions. One suspected function is fluid flow sensing- physical bending of cilia produces an influx of Ca^++^, which can then result in a variety of activated signaling pathways. Defective cilia and ciliary-associated proteins have been shown to result in cystic diseases. Autosomal Dominant Polycystic Kidney Disease (ADPKD) is a progressive disease, typically appearing in the 5^th^ decade of life and is one of the most common monogenetic inherited human diseases, affecting approximately 600,000 people in the United States. Because the mechanical properties of cilia impact their response to applied flow, we asked how the stiffness of cilia can be controlled pharmacologically. We performed an experiment subjecting cilia to Taxol (a microtubule stabilizer) and CoCl_2_ (a HIF stabilizer to model hypoxia). Madin-Darby Canine Kidney (MDCK) cells were selected as our model system. After incubation with a selected pharmacological agent, cilia were optically trapped and the bending modulus measured. We found that HIF stabilization significantly weakens cilia. These results illustrate a method to alter the mechanical properties of primary cilia and potentially alter the flow sensing properties of cilia.

## Introduction

Primary cilia are slender hair-like structures, present on most mammalian cells that protrude from the cell body into the extracellular space. Long considered vestigial structures, recent work has conclusively demonstrated that the primary cilium is in fact a calcium signaling center for the cell [1] organizing a large number of signaling pathways. Demonstrations that bending a primary cilium via fluid flow [2], optical tweezers [3], or micropipette [4] initiates intracellular calcium release imply that physiologically, the primary cilium is a flow sensor. However, the biological significance of this function remains unclear. While primary cilia have been identified as the key organelle in the pathogenesis of Autosomal Dominant Polycystic Disease (ADPKD) and there is evidence that altered flow sensing may contribute to ADPKD progression [5, 6], the *in vivo* relationship between fluid flow sensing by the cilia and kidney cyst formation and growth remains unclear, partly due to results supporting two independent disease hypotheses: the “two-hit model” [7] and the “futile repair model” [8]. For example, there is clear evidence [9, 10] that loss of cilia results in either stimulation or inhibition of cyst growth depending on the presence or absence of functional polycystins prior to loss of cilia.

In brief, the two-hit model primarily focuses on the role of defective polycystin gene products polycystin 1 and polycystin 2 [5] and polycystin related proteins PKD1L1 and PKD2L1 [1]. The futile-repair model accounts for similar features between ADPKD and renal injury responses by directly positing the flow sensing function of primary cilia and, when pathological function occurs, aberrant activation of a variety of cell responses follows. While both models enjoy experimental support, both models also suffer from unresolved objections, suggesting that some currently unidentified cilia-dependent signaling pathway may be required to promote cyst growth [11].

Our driving question here concerns the sensitivity of the sensor, which we show below is a combination of bending modulus EI and cilium length L. We have previously shown that exposure of ciliated cells to fluid flow changes the length of the cilium [12] and others have shown that cilia become elongated after injury [13–15], so we now examine the possibility of altering EI through pharmacological agents. Previous work has demonstrated a link between cilium length and hypoxia-inducible mechanisms [14], so we considered a similar approach to modifying the bending modulus.

Previous results have connected hypoxia to ciliary properties via the HIF-pVHL system. Ciliogenesis is regulated by pVHL [16], pVHL and glycogen synthase kinase-3β (GSK3β) together regulate cilium maintenance [17], and pVHL has been shown to act as a microtubule stabilizer [18]. Even so, it remains unclear if these functions of pVHL are HIF-regulation independent, as recent knockout experiments have shown that HIF-1α is not involved in renal cyst growth [19]. Regarding potential direct interactions between HIF and cilia, recent results show that prolyl hydroxylase inhibition of HIF-2α leads to HIF-2α accumulation within the cilia [20] and deubiquintination of HIF-1α is essential for ciliogenesis [21]. As expected, the majority of direct effects of hypoxia/hyperoxia on cilia have focused on motile cilia in the airway, where hyperoxia was shown to denude bronchial cells of motile cilia [22]. In [23] it was observed that hypoxic conditions stimulate the growth of cilia in *Tetrahymena thermophilae*. It is hoped that the results presented here call attention to the need for additional experiments that carefully assess any causal link between hypoxia and cilia.

Here, we consider the primary cilium as a passive mechanical structure capable of transducing kinetic energy of flowing fluid into elastic stress (bending) energy. Bending the cilium seems to be required to initiate calcium signaling [2], and the amount of bending in response to applied flow can be best characterized by the bending modulus [24]. We measured the bending modulus of primary cilia cultured in the presence or absence of the pharmacologic HIF stabilizer CoCl_2_ as well as taxol, a microtubule stabilizer, by optically trapping the distal end of the cilium and measuring the maximum distance the cilium tip could be displaced before breaking free of the optical trap. Measurements of the tip displacement, in combination with knowing how much force was applied by the optical trap, allows our computational model to output the bending modulus.

Because alterations to the bending modulus will result in altered ciliary bending in response to fluid flow, an essential element of the futile-repair model is the hypothesis that altering the mechanical properties of the cilium will result in altered flow-initiated ciliary signaling pathways. Primary cilium structure consists of 9 microtubule doublets anchored in the basal body, which itself is a highly organized structure comprising the centrosomes (microtubule triplets), transition fibers, a rootlet, and the basal foot [25] known collectively as the microtubule organizing center. We hypothesize that the mechanosensing function of cilia can occur by straining these microtubule structural elements in a similar manner to actin-mediated mechanosensation [26]. Preliminary results indicate that the basal body may have a role in differentiating mechanosensation from chemosensation [2, 27, 28]. The results presented here form an initial step towards clarifying the connection between cilia and cyst by determining and quantifying pharmacological-induced changes to the ciliary bending modulus. Once we know how to manipulate the mechanical properties of a cilium, we can design experiments to examine any causally-related altered flow responses.

Hypoxia is associated with the inability of pVHL to target hydroxylated HIF for degredation [29]. Consequently, an established alternative to performing cell culture within a hypoxia chamber is block hydroxylation of critical HIF prolyl residues with either Cobalt [30] or an iron chelator such as desferrioxamine [31].

Ciliary axonemes consist of 9 microtubule doublets, so the axoneme is susceptible to stabilization by Taxol [32]. Because the action of Taxol on microtubule mechanics is well known [32, 33], we used Taxol as a control.

## Results

### Cells tolerated pharmacologic treatments

**Fig 1:**
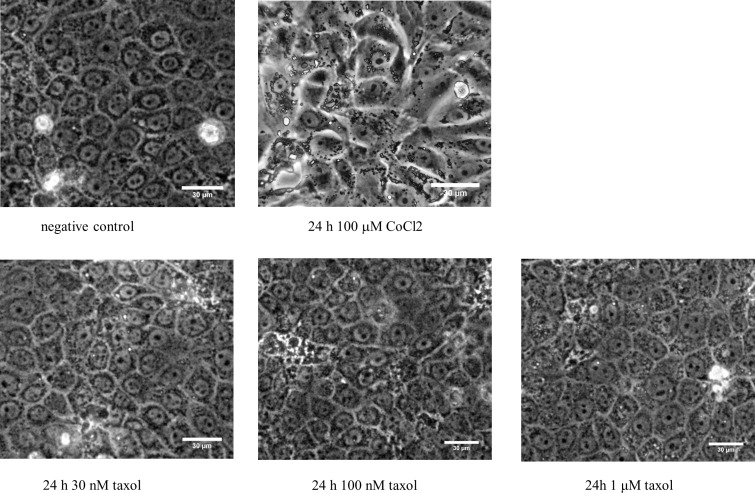
Live cell images of treated MDCK cultures. Images are of confluent ciliated MDCK monolayers at day 4: 3 days of serum starvation followed by 24 hours of media supplemented by the indicated pharmacologic agent. Images taken using a 10X phase objective, scale bars = 30 microns throughout. Cells treated with CoCl_2_ display altered morphology- cells appear larger and packing is relatively disorganized- but the monolayer remains confluent and cells express cilia. There was no evidence of increased apoptosis or shedding from the monolayer.

Fig 1 shows phase-contrast live cell images of MDCK monolayers subjected to our pharmacologic treatments. As seen in the images, the cells tolerated all drugs well, with no evidence of apoptosis, blebbing, or other maladaptive responses. CoCl_2_ does appear to modify tissue morphology, but there was no change in cell viability and the fraction of ciliated cells was indistinguishable from other cultures. In all cases, when serum was reduced for differentiation, intact tissue survived for 6 days before cells began to lift off the culture surface. Thus, we performed experiments on cells that had been serum starved for a total of 4 days.

### Cilium mechanical properties

**Fig 2:**
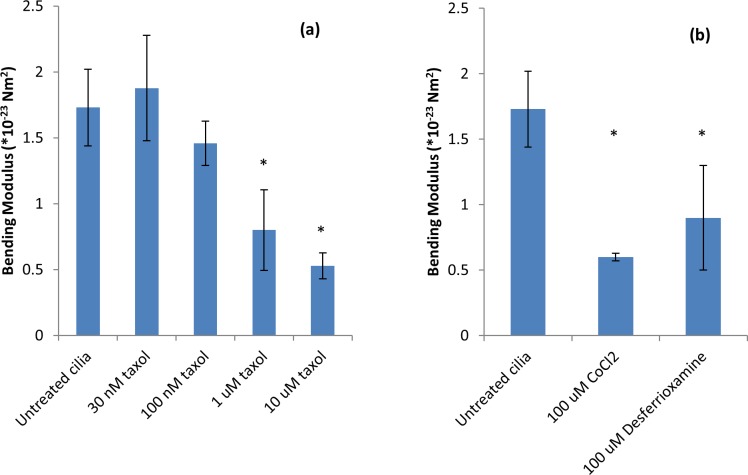
Bending modulus of cilia subjected to pharmacologic treatments. Asterisks indicate statistical significance p < 0.01 with respect to untreated cilia. Fig 2a shows that the bending modulus of cilia decreases in the presence of Taxol in a concentration-dependent manner. Fig 2b shows that addition of CoCl_2_ or desferrioxamine also causes cilia to be more flexible. Sample sizes: Untreated N = 6; 100 μM CoCl_2_ N = 3; 100 μM desferrioxamine N = 3.

Dynamic regulation of cilia length is an essential adaptive response to fluid flow [12, 34] yet its regulation is not well characterized in vertebrates. Signaling pathways known to be involved with dynamic control of cilium length include cAMP-dependent protein kinase A (PKA) activation [34, 35] and *ros* cross-hybridizing kinases (RCKs) [36]. While measurements of cilium lengths are subject to fixation errors [37] as many fixatives grossly deform cilia morphology, few reports of measured cilium lengths using live cilia exist. In contrast to reports showing fixed cilia are of uniform (or nearly uniform) length, we observed that live cilium lengths within a single culture varied widely, ranging from 4 μm to longer than 25μm, in agreement with an earlier careful study [38]. Although we measured the length for each of the trapped cilia (sample size N ranged between 3 and 6, see Fig 2), we intentionally selected cilia with similar lengths (between 4 and 6 μm) to avoid potential confounding effects. Because multiple studies indicate cilium length alterations occur in a variety of contexts, the issue of cilium length measurements should be re-visited.

#### Cilium mechanical properties are independent of culture support

*In vivo*, epithelial tissue is polarized and generates directed transport of salt and water. Epithelial tissue grown on impermeable supports cannot transport fluid and studies using MDCK cultures occasionally report the formation of ‘domes’, most likely created when the transported fluid lifts tissue off the support. We wanted to check for any possible confounding effect due to growing cells on impermeable supports. Our data (not shown) indicates that cells grown on impermeable supports (plastic or glass) do not express cilia with mechanical properties different from cells cultured on permeable supports.

#### Cilium mechanical properties are independent of measurement method

The bending modulus of untreated cilia was separately determined by two methods; the deformed cantilever and beam buckling methods (see Methods section). For the deformed cantilever method, we obtained a bending modulus EI = (1.73 ± 0.29) *10^−23^ Nm^2^ while for the beam buckling method we obtained a bending modulus EI = (1.96 ± 0.13) *10^−23^ Nm^2^. As expected, both methods yield the same bending modulus.

#### Cilium mechanical properties vary with biochemical control

By comparing the applied force from the optical trap to the cilium tip deflection the bending modulus may be calculated from Eq 7 (below). Fig 2 presents our results. As expected, addition of Taxol lowers the bending modulus in a concentration-dependent manner as reported elsewhere [32, 33]. Similarly, addition of CoCl_2_ lowered the bending modulus, but it is not clear from our data if the action of CoCl_2_ with respect to Taxol is independent or compensatory.

We wished to determine if the weakening of the primary cilium was due to Cobalt, independent of HIF, so we incubated a culture in the presence of 100 μM desferrioxamine and measured the bending modulusImage analysis of desferrioxamine-treated cilia buckled with the optical trap results in EI = 0.9 ± 0.2*10^−23^ Nm^2^, similar to CoCl_2_ data.

## Discussion

Our effort to determine the mechanical properties of the primary cilium is based on the idea that the primary cilium is a mechanical flow sensor, the mechanical response of the cilium to fluid shear is thus a measure of the sensor sensitivity. The importance of ‘sensor sensitivity’ is based on the idea that flow sensing by the cilium is an essential regulatory element of overall cell, tissue, and organ function. Understanding ways to control or otherwise alter the sensor sensitivity can then be viewed as a potential therapeutic activity, both for ciliopathies and injury recovery.

Cilium mechanical properties were primarily determined by modeling the cilium as a modified cantilever as shown in the ‘Methods’ section. By measuring the force applied by an optical trap to the cilium tip, the corresponding tip displacement, and the cilium length, the bending modulus can be determined. Comparison of our untreated cilia with previous results [24, 39] shows we are in agreement. Similarly, comparing our results with previous work applying Taxol to microtubules [33, 40] shows agreement.

The results presented here are to be primarily viewed within the context of a broader effort to develop therapeutic strategies for ADPKD, which to date remains non-treatable. Our approach is based on the futile-repair hypothesis and considers fluid conditions present within a kidney cyst or ruptured tubule as a causal agent. In addition to containing elevated levels of inflammatory cytokines [41], cyst fluid is stagnant and the tissue hypoxic [42]. Similarly, fluid within a ruptured tubule or within an explanted organ is stagnant and the environment hypoxic. Therefore, treatment of Acute Kidney Injury (AKI) is a second potential application for this work, as models have shown [13] that the cilium length is altered in AKI. A third potential application is the development of improved perfusion protocols to improve kidney transplant success rates by preventing Ischemia Reperfusion Injury [43]. Indeed, recent results [15] show that removal of one kidney results in increased levels of reactive oxygen species and elongated cilia in the remaining kidney. This is relevant because hypoxia can be associated with increased levels of reactive oxygen species, although the literature [44–46] indicates the connection is still unclear. Our driving question concerned the sensitivity of the primary cilium as a fluid flow sensor, which is a combination of bending modulus EI and cilium length L. It is known that exposing ciliated cells to fluid flow shortens the cilium and that cilia become elongated after cessation of fluid flow, so we examined the possibility of altering EI as an additional sensor parameter. Because it is known that hypoxic conditions induce cilia to lengthen, we selected pharmacological agents that act to stabilize HIF and have determined that when HIF is stabilized, EI decreases. While it is possible that the pharmacological compounds themselves could be responsible for the increased flexibility, a more likely explanation is that the mechanical properties of the primary cilium are somehow regulated as part of the HIF-pVHL system. Indeed, pVHL has been shown to stabilize microtubules [17, 18], but there is evidence that the stabilizing function of pVHL function is HIF-independent [17], implying additional experiments are required to clearly demonstrate a causal link between hypoxia and ciliary alternations.

In the context of our overall research program, our results demonstrate that the bending modulus can be altered pharmacologically, providing an additional degree of freedom in our studies of primary cilium function. We can now independently control cilium length (via flow) and bending modulus (via pharmacology), expanding quantitative investigations of flow sensitivity by the primary cilium and determining a rational basis for the flow sensing function of primary cilia.

## Sources of Error

### Cilium length ‘L’

Cilium lengths were measured on live cells, avoiding the well-documented degradation that occurs during chemical fixation [37]. Even so, the length determination depends on the depth of focus of the microscope objective, which is ± 0.4 μm. The uncertainty in length creates uncertainty in our analytical model, which scales as L^3^, and as we will show, this uncertainty is the largest source of error. The fractional uncertainty in L (ΔL/L) creates up to 30% fractional uncertainty in EI, which is largest for the shortest cilia used (4 μm) in this study.

### Applied trap force ‘F’

The QPD data itself presents approximately 10% uncertainty in the applied force. However, because our data processing algorithm [47], while insensitive to the shape of a trapped object, was checked only for free objects, the fact that trapped cilia are anchored at one end could potentially invalidate our data processing method. We checked this by applying the trap to different locations along the ciliary axoneme. With the cilium in a neutral position (oriented vertically), the trap was applied to various and QPD data acquired. The calculated spring constant (applied force) did not vary more than 10%, and so we are confident that our data processing algorithm provides valid force data.

## Conclusions

Cilium mechanical properties were determined by modeling the cilium as a modified cantilever as shown above. By measuring the force applied by an optical trap to the cilium tip, the maximum tip displacement, and the cilium length, the bending modulus can be determined. Comparison of our untreated cilia with previous results [24, 39] shows we are in agreement. Similarly, comparison with previous work applying taxol to microtubules shows a general softening trend, as shown previously [33, 40].

Our primary finding is that cilia expressed by HIF-stabilized tissue are more flexible as compared to wildtype. Analysis shows that more flexible cilia should be associated with longer cilia, in agreement with previous observations. We thus conclude that the cell may regulate the length of a cilium to maintain a constant ‘setpoint’ of sensor sensitivity.

## Methods

### Analytical model of primary cilium

#### Deformed cantilever

Following [39, 48], we model the cilium as a 1-D nonlinear uniform cantilevered beam. This basic model for a primary cilium treats a cilium as a homogeneous flexible cylindrical beam, anchored at the basal end and free to move at the distal end. Because primary cilia, unlike motile cilia, do not actively generate internal forces, we can model the primary cilium in terms of a passive beam: there is no generation of forces and/or moments within the cilium. Because the slenderness (length/diameter) of the cilium is large, we may neglect both rotatory inertia and transverse shear and approximately describe the cilium shape in terms of a 1-D object, the so-called neutral axis [49]. Under the conditions described above, the time-independent shape Y(s) of the neutral axis of a cilium is given by the linearized Euler-Bernoulli law for pure bending:

$$EI\frac{\partial^{4}Y}{\partial s^{4}}=w$$ (1)

Where ‘w’ is the externally applied distributed force per length, ‘E’ is the Young’s modulus of the cilium, ‘I’ the area moment of inertia (for a cylinder of radius ‘a’, I = πa^4^/4), and EI together referred to as the ‘flexural rigidity’ or ‘bending modulus’, having units of Force*area.

Because our measurement method creates static deformations, there is no fluid drag acting on the cilium (w = 0). The relevant boundary conditions for the fixed (basal) end and free end are:

1. The fixed end cannot move:
  Y(0)=0 (2)
2. The fixed end has a bending moment modeled as a nonlinear spring:
  $$\frac{d^{2}Y(0)}{ds^{2}}-\frac{L}{EI}\left(k\frac{dY(0)}{ds}+\alpha {\left(\frac{dY(0)}{ds}\right)}^{2}\right)=0$$ (3)
3. At the free end (s = L), the bending moment vanishes:
  $$\frac{d^{2}Y(L)}{ds^{2}}=0.$$ (4)
4. The free end is subject to a shear load from the optical trap:
  $$\frac{d^{3}Y(L)}{ds^{3}}=-\frac{F_{trap}}{EI}.$$ (5)

The Euler-Bernoulli equation with these boundary conditions has a closed-form solution:

$$Y(s)=\frac{s[sF_{trap}(3L-s)\alpha -3\;EI(k+\sqrt{k^{2}+4F_{trap}\alpha })]}{6\;EI\;\alpha }$$ (6)

With corresponding tip displacement:

$$Y(L)=\frac{L[2L^{2}F_{trap}\alpha -3\;EI(k+\sqrt{k^{2}+4F_{trap}\alpha })]}{6\;EI\;\alpha }$$ (7)

and basal curvature, corresponding to the developed local elastic stress energy:

$$\frac{d^{2}Y(0)}{ds^{2}}=\frac{L}{EI}F_{trap}$$ (8)

Eq 7 is used to calculate the bending modulus. When the cilium escapes the optical trap, the cilium tip reached the maximum displacement Y(L) and the restoring force of the bent axoneme is equal to the applied trap force. Table 1 provides a summary of the parameters used in this model.

**Table 1:** Values of model parameters.

Model parameter	Value
Cilium length L	As per measurement
Applied trap force F_trap_	As per measurement
Maximum tip displacement Y(L)	As per measurement
Bending modulus EI	What is being determined
Linear rotatory spring constant k	4.6 *10^−12^ N/rad
Nonlinear rotatory spring constant α	-1.0 *10^−10^ N/rad^2^

#### Beam buckling

**Fig 3:**
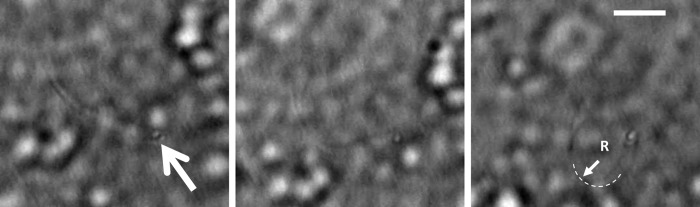
Application of the optical trap to induce buckling of an untreated cilium. Scale bar = 5 microns, arrow indicates location of the optical trap. Rightmost panel indicates radius of curvature R.

An independent method of determining the bending modulus uses the optical trap to apply a compressive force to the cilium, rather than a shearing force as described above. Application of a compressive (buckling) force [50] provides an independent method to estimate the bending modulus [50] and relies on fewer parameters than application of a shearing force. A slender filament will bend in response to a compressive force F = EI/2R^2^, where EI is the bending modulus and R is the radius of curvature of the bend (see, for example, [51]). Application of the optical trap to the distal end of a cilium then moving the trap towards the basal end results in compressive bending of the cilium (see Fig 3). The bending radius is measured from the acquired image, and when combined with the calculated applied force, the bending modulus is obtained.

Fig 3 presents images of the beam buckling method used to calculate the bending modulus.

### Cell culture

Experiments were carried using the MDCK cell line (American Type Culture Collection CCL-34), free from contamination and from young stock. Cells were maintained on several different substrates: collagen coated 35mm diameter glass bottom petri dishes (MatTek Corporation, Ashland MA, product # P35G-1.5-14-C), 60mm plastic petri dishes (Celltreat products, product # 229660, tissue culture treated), and collagen-coated Millicell-CM inserts (inner diameter 25 mm, permeable support area 0.6 cm^2^; Millipore Corp, Billerica, MA). Cells were grown and maintained at 37°C, 5% CO_2_. Growth medium consisted of the following (final concentrations): Dulbecco's Modified Eagle Medium w/o glucose and Ham’s F12 at a 1:1 ratio, 5 mM glucose, 15 mM 4-(2-hydroxyethyl)-1-piperazineethanesulfonic acid (HEPES), 0.06% NaHCO_3_, 2 mM L-glutamine, 10% fetal bovine serum (FBS). For differentiation, FBS was reduced to 1%. The amount of medium was restricted so that the apical fluid was thin enough to allow sufficient O_2_ to diffuse to the monolayer.

### Pharmacologic treatments

After 3 days of serum starvation, some cultures were incubated for 24 hours in media supplemented with 100 μm CoCl_2_, 100 μm desferrioxamine, or taxol (various concentrations). CoCl_2_ was supplied by MP Biomedicals as cobalt chloride hexahydrate, in crystalline form. Taxol and desferrioxamine were supplied by Santa Cruz Biotechnology (Dallas, TX), certified >99% pure, in solid form.

### Microscopy

Imaging and manipulations of terminally differentiated epithelial monolayers were carried out using a Leica DM 6000 upright microscope equipped with a heated and CO2 controlled incubation chamber (Solent Scientific). The microscope stage (Prior Scientific H30XY2) was accurate to ±0.04 μm. Brightfield image acquisition and optical trap monitoring were performed by a Point Grey Instruments ‘Flea’ digital video-rate camera.

### Optical tweezers

As described elsewhere, the source for the single-beam 3D trap was a Crystalaser diode-pumped Nd:YAG continuous-wave single mode laser providing 0.5W optical power from a 10W electrical power supply. The optical tweezer was constructed using Qoptiq Microbench® optomechanical mounts. The objective lens used was a Leica 63X NA 0.9 U-V-I HCX long working distance plan apochromat dipping objective with a 2.2 millimeter working distance. The tweezer couples into the microscope through an existing lateral port. A side-looking dichroic mirror (Chroma) mounted within the fluorescence turret provides the ability to perform normal microscope viewing while the tweezers are operating. The fixed-position optical trap has a beam waist 0.3 μm and Rayleigh length 0.4 μm.

Objects held within the trap diffract the trapping beam. The spatial dynamics of the diffracted beam were recorded using a quadrant photodiode (QPD) and the data analyzed as per [47].

**Fig 4:**
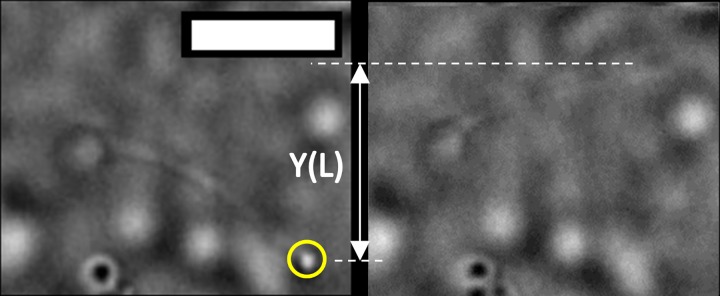
Optically trapping and bending a cilium exposed to 100 μM CoCl_2_ for 24 h. Circle identifies location of the trap. The right panel shows the cilium at maximum deflection Y(L), the left shows the cilium after it escaped from the trap and has fully returned to its equilibrium configuration.

**Fig 5:**
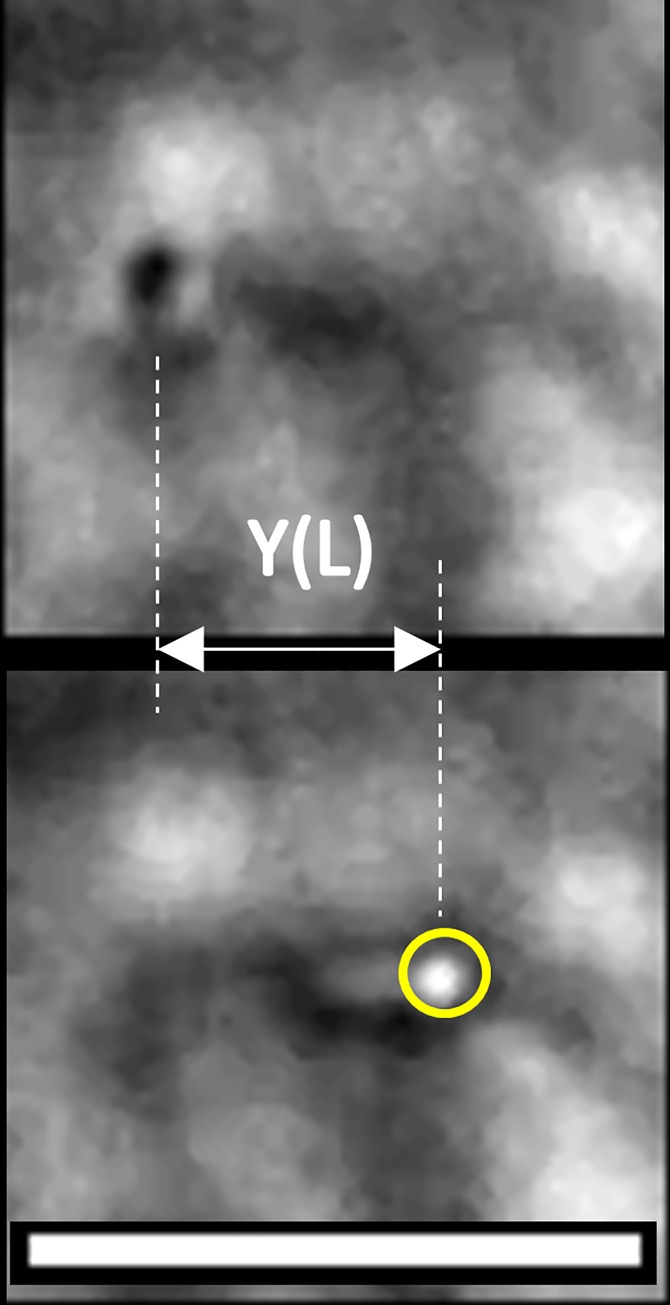
Optically trapping and bending a cilium exposed to 10 μM Taxol for 24 h. Scale bar = 3 microns. Circle identifies location of the trap. Cilium is oriented along the optical axis of the microscope, in contrast to the cilium in Fig 4. Bottom panel shows the cilium at maximum deflection Y(L), top panel shows the cilium after it escaped from the trap and has fully returned to its equilibrium configuration.

Applying the trap to a primary cilium proceeded as follows. First, the trap location was precisely determined by trapping a small piece of floating cell debris. Turning the trapping laser off and using brightfield illumination, a cilium was moved to the location of the trap and focus adjusted to align the trapping plane to the cilium tip. The optical trap was turned on and QPD data acquired for several seconds. After QPD data acquisition, the Flea camera acquired video (30 fps) and the stage slowly laterally translated the cilium until it broke free of the trap (See Figs 4 and 5). The final displacement was recorded, the trap turned off, another cilium moved into position, and the procedure repeated. Each trapped cilium yielded approximately 6 independent measurements of F_trap_,

### Statistical Analysis

Data is presented with +/- 1 standard deviation error bars.

Statistical analysis was done using an online one-way ANOVA with post-hoc Tukey HSD test calculator [52]. Values of p < 0.01 are considered significant.

## Supporting Information

S1 File Table A. Numbers and provenance data for trapped cilia. Data includes: Applied trap force (‘F’), in units of pN; lateral displacement of trap location from untrapped axoneme (‘d’), in units of microns; best fit bending modulus (‘EI’), in units of N*m^2^.
(DOC)
