# Supplementary material for: HIF Stabilization Weakens Primary Cilia
Source: PLoS One. 2016 Nov 3;11(11):e0165907. doi: 10.1371/journal.pone.0165907 (PMC5094786; doi:10.1371/journal.pone.0165907)
Supplement: S1 File — Table A. Numbers and provenance data for trapped cilia. Data includes: Applied trap force (‘F’), in units of pN; lateral displacement of trap location from untrapped axoneme (‘d’), in units of microns; best fit bending modulus (‘EI’), in units of N*m2. (DOC) [file pone.0165907.s001.doc]

**Supporting Information File S1**

**HIF stabilization weakens primary cilia**

A. Resnick

Table A. Numbers and provenance data for trapped cilia. Data includes: Applied force (‘F’), in units of pN; lateral displacement of trap location from untrapped axoneme (‘d’), in units of microns; best fit bending modulus (‘EI’), in units of N*m^2^.

|  | F | d | EI*10-23 (fit) | |  | |  |  |  | |  |  |  |
| --- | --- | --- | --- | --- | --- | --- | --- | --- | --- | --- | --- | --- | --- |
| untreated | 8.520809 | 1.35 | 1.359 |  | |  |  |  |  | |  |  |  |
| L = 4 | 9.681107 | 1.19 | 1.66 |  | | avg | 1.755667 |  |  | |  |  |  |
|  | 11.62247 | 0.97 | 2.21 |  | | stdev | 0.296214 |  |  | |  |  |  |
|  | 11.0047 | 1.36 | 1.69 |  | |  |  |  |  | |  |  |  |
|  | 10.59831 | 1.03 | 1.975 |  | |  |  |  |  | |  |  |  |
|  | 10.31617 | 1.31 | 1.64 |  | |  |  |  |  | |  |  |  |
|  |  |  |  |  | |  |  |  |  | |  |  |  |
| CoCl2 | 9.477849 | 4.2 | 0.598 |  | | avg | 0.598333 |  |  | |  |  |  |
| L=4 | 9.3813 | 3.88 | 0.635 |  | | stdev | 0.036501 |  |  | |  |  |  |
|  | 4.432846 | 1.95 | 0.562 |  | |  |  |  |  | |  |  |  |
|  |  |  |  |  | |  |  |  |  | |  |  |  |
| 30 nm taxol | 7.497135 | 0.58 | 2.07 |  | |  |  |  |  | |  |  |  |
| L =4 | 9.08 | 1.04 | 1.72 |  | | avg | 1.884 |  |  | |  |  |  |
|  | 7.061201 | 0.65 | 1.85 |  | | st dev | 0.137405 |  |  | |  |  |  |
|  | 7.297322 | 0.71 | 1.81 |  | |  |  |  |  | | EI | error | t-test wrt control |
|  | 6.001497 | 0.45 | 1.97 |  | |  |  | Untreated cilia | | | 1.73 | 0.29 |  |
|  |  |  |  |  | |  |  | 30 nM taxol | | | 1.884 | 0.137405 | 0.001171185 |
| 100 nM taxol | 14.38952 | 2.06 | 1.59 |  | |  |  | 100 nM taxol | | | 1.46 | 0.168226 | 0.100616029 |
| L = 4 | 16.65067 | 3.22 | 1.27 |  | | avg | 1.46 | 1 uM taxol | | | 0.8 | 0.305123 | 0.010870274 |
|  | 12.19782 | 1.785 | 1.52 |  | | stdev | 0.168226 | 100 uM CoCl2 | | | 0.6 | 0.03 | 0.00016538 |
|  |  |  |  |  | |  |  |  | | |  |  |  |
| 1 uM taxol | 5.983701 | 6.96 | 0.79 |  | |  |  |  | |  | | | |
| L =6 | 6.067812 | 11.45 | 0.505 |  | | avg | 0.801667 |  | | | | | |
|  | 6.453566 | 5.13 | 1.11 |  | | stdev | 0.302669 |  | | | | | |
